# Analysis of frequency domain features for the classification of evoked emotions using EEG signals

**DOI:** 10.1007/s00221-025-07002-1

**Published:** 2025-02-14

**Authors:** Samannaya Adhikari, Nitin Choudhury, Swastika Bhattacharya, Nabamita Deb, Daisy Das, Rajdeep Ghosh, Souvik Phadikar, Ebrahim Ghaderpour

**Affiliations:** 1 Department of Information Technology, Gauhati University, Gopinath Bordoloi Nagar, Jalukbari, Guwahati, Assam 781014 India; 2 Department of Computer Science and Engineering, Indraprastha Institute of Information Technology Delhi, Okhla, New Delhi 110020 India; 3 Pandit Deendayal Upadhyaya Adarsha Mahavidyalaya, Amjonga, Goalpara, Assam 783124 India; 4 Center for Translational Research in Neuroimaging and Data Science (TReNDS), 55 Park Pl NE, Atlanta, GA 30303 USA; 5 Department of Earth Sciences, Sapienza University of Rome, Piazzale Aldo Moro, 5, Roma, RM 00185 Italy

**Keywords:** Electroencephalogram (EEG), Emotion classification, Frequency domain analysis, Intrinsic mode function (IMF), Machine learning, Variational mode decomposition (VMD)

## Abstract

Emotion is a natural instinctive state of mind that greatly influences human physiological activities and daily life decisions. Electroencephalogram (EEG) signals created from the central nervous system are very useful for emotion recognition and classification. In this study, EEG signals of individuals are analyzed by the variational mode decomposition (VMD) for frequency domain features to recognize visual stimuli-based evoked emotions (happy, sad, fear). After cleaning EEG signals from artifacts, VMD is employed to decompose the signal into its respective intrinsic mode functions (IMFs). A sliding windowing approach is adopted to calculate the power distributions in each of the predefined frequency bands. The results reveal that extracting frequency domain features using a sliding window of 3 s significantly enhances the efficiency of analyzing induced emotions in subjects. The random forest model shows promising results in classifying various emotions, achieving an accuracy of 99.57% for validation and 99.36% for testing. Moreover, it is observed that the fifth IMF has a strong relationship with emotion elicited from visual stimuli. In addition, the features of the trained model are analyzed by Shapley additive explanations.

## Introduction

Emotion recognition has emerged as a pivotal element in the realms of human–computer interaction. As machines continue to become a prevalent part of our daily routines, the ability to comprehend and interpret human emotions becomes crucial in elevating the overall human–computer interaction experience. Various modalities, including facial expressions, speech, and physiological signals, have been explored for emotion recognition in literature (Black and Yacoob 1997; Petrushin 1999; Brosschot and Thayer 2003; Kim et al. 2004; Wagner etal. 2005; Anderson and McOwan 2006). Electroencephalography (EEG) has garnered a great interest compared to other methods because of its special capacity to obtain information directly about the central nervous system (Arbour 2003). EEG is a sophisticated non-invasive neuro-imaging method used to monitor the brain’s electrical activity. Through strategically placed electrodes on the scalp, EEG captures neural oscillations, revealing real-time information about the brain’s functioning. By measuring the fluctuations in voltage caused by electric activity in neurons, EEG not only records the brain’s electrical output but also provides a deeper understanding of its cognitive processes, emotional reactions, and neurological health.

Visual stimuli in EEG experiments involve presenting images or visual content to participants, aiming to evoke specific emotional responses. These responses are recorded with an EEG device reflecting neural reactions to these visual stimuli, offering valuable insights into the intricacies of emotion processing and cognitive engagement. The study of emotion through EEG signals involves capturing the brain’s electrical activity, which responds to different emotional states (Coan and Allen 2004; Li etal. 2009; Petrantonakis and Hadjileontiadis 2011). Previous research has utilized EEG to classify emotions induced by visual stimuli, such as videos and images (Murugappan et al. 2009). Analyzing EEG signals in identifying emotions is not only beneficial for effective human–computer communication, but also valuable to understand the emotional connections in the brain.

Variational mode decomposition (VMD) has been proven to be a versatile signal processing technique, particularly in non-homogeneous signal analysis. Originating from the work by Dragomiretskiy and Zosso (2013), VMD has excelled in diverse applications, such as denoising electrocardiogram (ECG) signals and identifying focal activity in EEG signals (Taran and Bajaj 2018). The non-recursive nature of VMD sets it apart from other methods, ensuring resilience against noise and sampling rate variations. Significant advancements have been made in the area of emotion classification utilizing EEG signals, provided insights into the applications and understanding of brain computer interfaces. The following section provides a brief summary of the related literature of EEG, data acquisition, data preprocessing, feature extraction, and classifications.

### Related work

Wang et al. (2014) investigated diverse EEG features for classification of emotion. They emphasized the effectiveness of power spectrum features and the efficacy of a linear dynamic system-based feature smoothing method. Their method yielded notable accuracy, providing a way for visualizing emotion changes. Li and Lu (2009) concentrated on classifying emotions like happiness and sadness through facial expressions and utilized Gamma-band EEG signals. Their method, incorporating common spatial patterns and linear-SVM, demonstrated high accuracy of 93.5% and 93.0% for trials of 3 s and 1 s, respectively. Dabas etal. (2018) utilized Naïve Bayes and support vector machine (SVM) models to classify emotions based on EEG data, achieving accuracy of 78.06% and 58.90%, respectively. Considering factors, such as arousal and valence, provided insights into participants’ emotional states while watching musical videos, opening avenues for personalised recommendations based on emotional states. Mehmood and Lee (2015) conducted research on emotion identification from EEG brain waves using SVM and k-nearest neighbor (KNN) classification methods. In their experiment, four different emotional stimuli were presented to each of the five male subjects chosen. The preprocessing of EEG raw data involved using Hjorth parameters for feature extraction across all channels at each epoch. The remarkable finding in their study was that the brain signals of EEG can detect emotions. These developments built on previous studies contribute to a better understanding on classification of emotions with EEG, thus opening new avenues for further research in this field.

A multidimensional feature extraction model using wavelet packet decomposition (WPD) and VMD was introduced by Zhang etal. (2020). They demonstrated that their model has higher competitiveness and universality, overcoming the limitations of existing decomposition algorithms and enhancing the field. Alhalaseh and Alasasfeh (2020) applied empirical mode decomposition (EMD) and VMD filters to clean EEG signals and further classified the emotions from EEG signals using entropy and Higuchi’s fractal dimension as features. Pandey and Seeja (2022) proposed a subject-independent emotion detection algorithm with VMD as feature extractor and deep neural network (DNN) as classifier. This group of research contributes towards the direction in which emotion recognition systems are moving nowadays, suggesting various ways of developing this field.

Diverse time-frames, duplicate channels, and individual variances in EEG signals pose hurdles to the identification of emotions in EEG data. Li et al. (2024) proposed a cross-attention-based dilated causal CNN with domain discriminator method to solve these problems. Their method works better than state-of-the-art approaches in multi-view EEG-based emotion detection, according to testing on the SEED and DEAP datasets. Guo and Wang (2024) proposed a convolutional gated recurrent unit-driven multidimensional dynamic graph neural network to address the issue of separating temporal and spatial feature extraction in EEG-based emotion recognition models. Their model collects spatial and temporal information, including non-Euclidean spatial aspects, by utilizing multidimensional dynamic graph convolution and merging convolution layers, producing competitive outcomes in subject-independent emotion recognition tasks.

### Main contributions

The present study highlights the application of VMD to analyze EEG signals for emotion recognition, offering a significant improvement over conventional techniques, such as EMD and Fourier-based methods. Unlike EMD, VMD provides a more robust and non-recursive decomposition, which is advantageous in maintaining stability across varying signal characteristics and minimizing the impact of noise. The use of VMD allows for a more precise extraction of intrinsic mode functions (IMFs), which retain critical frequency-domain features associated with different emotional states. This level of decomposition aids in identifying subtle signal changes that are essential for accurate emotion classification, enhancing the efficacy of the emotion recognition task. Therefore, the main contributions of this research include:

- Highlighting the potential of VMD to recognize visual stimuli based evoked emotions from EEG in its temporal domain.
- Investigating which IMFs have the most significant relationship with emotion elicited from visual stimuli.
- Comparing four popular machine learning models for EEG emotion classification and employing Shapley additive explanations (SHAP) to establish the relationship between model predictions and input features.

## Materials and methods

**Fig. 1:**
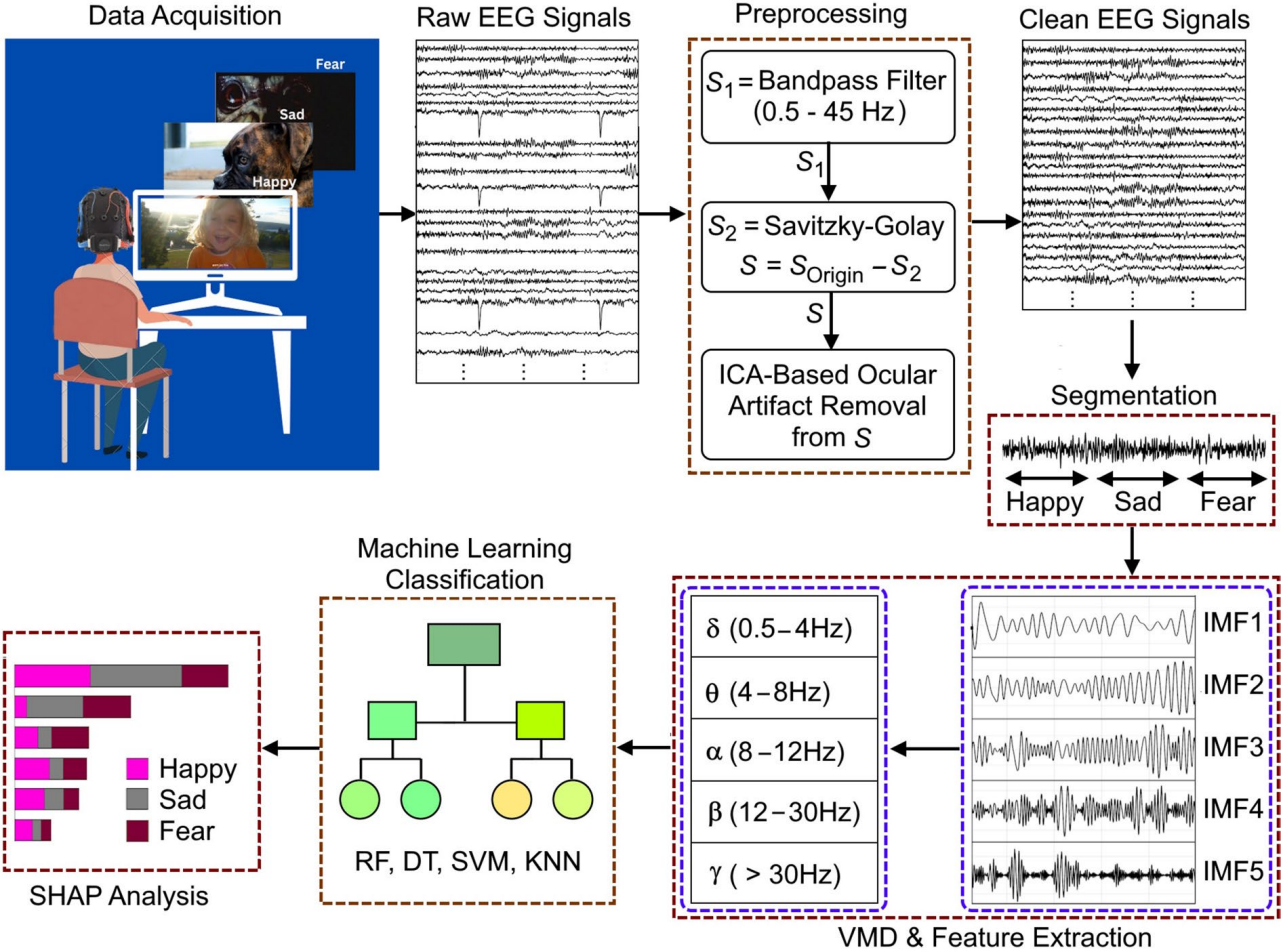
Workflow of this study

Figure 1 shows the flow diagram of the research methodology enclosing the data acquisition protocol, frequency based feature analysis, classification using four popular machine learning models, and the interpretability of the trained model.

### Data collection

**Fig. 2:**
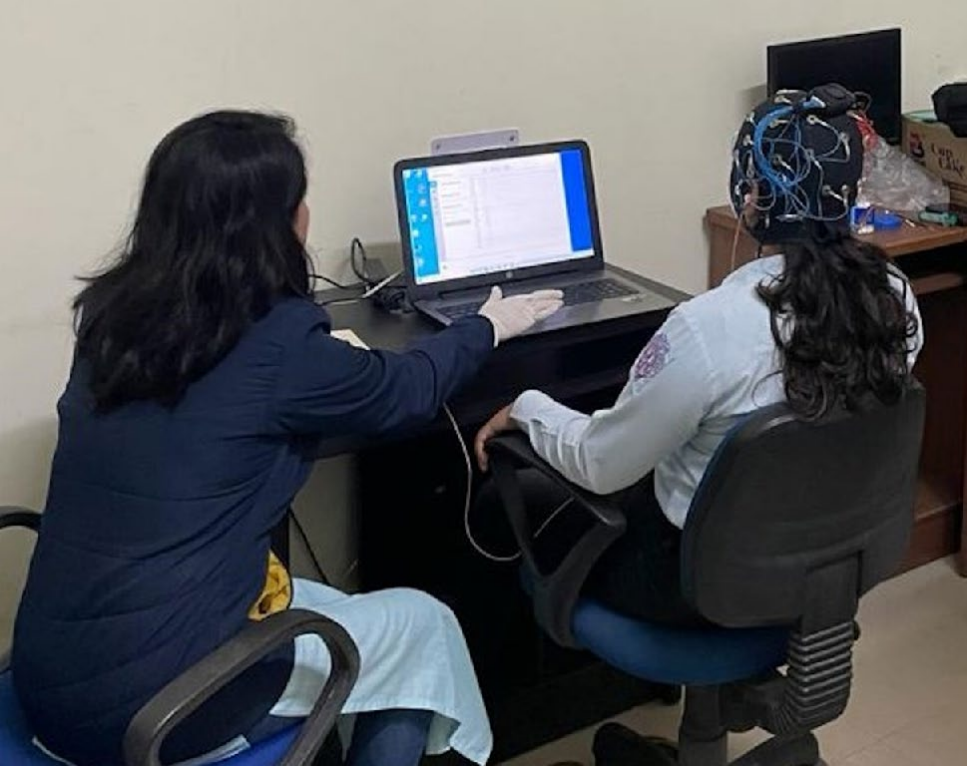
Data collection

The main aim of the experiment is to analyze the brain activity of individuals for different induced emotions and to classify them depending on their EEG signals. For the purpose, a total of 20 healthy participants aged between 18 and 23 years from the Department of Information Technology, Gauhati University, Guwahati, India, were initially considered, see Fig. 2. However, only 10 participants, comprising 1 female and 9 male, are considered for the final analysis based on the emotional feedback.

In the experimental protocol, visual stimuli were considered to elicit various emotions in the participants. For the purpose, the well-annotated images for the visual emotions (happy, sad, and fear) from the “Open Image Dataset v7 and Extensions" were considered. This is a widely recognized resource that has been leveraged in prior research for emotion induction tasks (Chechik 2017; Kuznetsova et al. 2020). The 32-electrode Emotiv Epoc Flex gel kit from Emotiv Inc. was used for collecting the EEG data, following the 10–20 standard for electrode placement with sampling frequency of 128 Hz. The selected channels for recording brain activity were Fp1, F7, F3, FC1,CZ, FZ, C3, FC5, FT9, T7, CP5, CP1, P3, P7, PO9, O1, OZ, PZ, O2, PO10, P4, P8, CP2, CP6, T8, FT10, FC6, C4, Fp2, F4, F8, and FC2 with common mode sense (CMS) and driven right leg (DRL) as references. During EEG recording, CMS and DRL electrodes were used as references. The CMS electrode was placed at P3 (left mastoid), and the DRL at P4 (right mastoid). This setup, standard in Emotiv headsets, provides stable differential recordings by floating the reference on the common mode body potential.

**Fig. 3:**
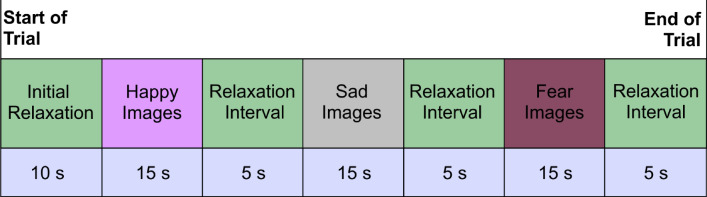
Trial recording paradigm

The data recording pipeline of a single trial is demonstrated in Fig. 3. Participants are initially instructed to relax for 10 s, during which they viewed a white screen presenting the word “Relax” to ease into the experiment. Following this, a series of three images, each shown for 5 s, aimed to induce emotion of happiness. This kind of sequence has been repeated for “sad” and “fear” stimuli respectively interspersed with 5-s relaxation intervals in between emotions. After an additional 10-s relaxation period, Phase 2 commenced. Similar to Phase 1, Phase 2 involved a different set of visual stimuli, allowing for diverse emotional states while maintaining consistent data recording across trials and participants. Verbal feedback from participants has been taken immediately after each experimental phase to determine whether they experienced the intended emotion. Based on the verbal feedback, participants whose reported emotions did not align with the annotated labels were excluded from the study. This measure was implemented to ensure that only data from subjects who genuinely experienced the target emotion were analyzed. This verbal feedback process served as a self-report validation tool, providing confidence that the emotional states evoked matched those intended.

During the recording participants were asked to minimize their movement in order to minimize the different artifacts and noises. However, the subjects were not restricted from natural shifts. This flexibility was intended to balance the need for high-quality EEG recordings with participants’ comfort. To mitigate potential arousal from the request to remain still, multiple relaxation periods were incorporated throughout the experiment. These breaks helped participants maintain a relaxed state, reducing the potential for inhibitory effects on emotional induction. An informed consent was signed by each of the participants before starting of the experiment.

### Data preprocessing

Raw EEG data is often combination of information and artifacts. To remove those artifacts and noises and extract the relevant information from the unfiltered EEG, the data need to be preprocessed (Phadikar et al. 2022; Ahmed et al. 2023). The preprocessing pipeline that is followed consists of three primary steps, namely, bandpass filtering, Savitzky–Golay based artifact removal, and independent component analysis (ICA) based ocular artifact removal (Li et al. 2006).

In the first step, a bandpass filter operating in the range of 0.5 Hz (lower bound) to 45 Hz (upper bound) has been implemented to focus on the relevant frequency bands, i.e., Delta (0.5–4 Hz), Theta (4–8 Hz), Alpha (8–12 Hz), Beta (12–30 Hz), and Gamma (30 Hz and above). The filtering was implemented using a cascade of low-pass and high-pass finite impulse response (FIR) filters. The filtering was performed using the zero-phase forward-backward filtering to ensure linear phase response and prevent phase distortion.

In the second step, Savitzky–Golay smoothing filter is implemented with a frame length of 127 and an order of 5 to the band-pass filtered data. The average trend in the EEG signals is eliminated by subtracting the reference signal, produced using the Savitzky–Golay smoothing filters from the EEG signals (Ghosh et al. 2022).

In the third step, ICA is employed to mitigate the influence of ocular artifacts on the data (Jutten and Herault 1991). The ICA is implemented using the “runica” function from the EEGLAB toolbox. The independent components are inspected visually using EEGLAB’s tools to identify those exhibiting characteristics of eye artifacts. The artifactual components are selected and excluded, retaining only the non-artifactual components. The information from the EEG recordings with minimal artifact are now recovered, ready for further analysis.

### Feature extraction

Variational mode decomposition (VMD) is a powerful technique in signal processing that dissects complex signals into their inherent components, known as intrinsic mode functions (IMFs). Unlike fixed Fourier transforms or wavelets, VMD uses a variational optimization approach to capture non-stationary and highly oscillatory signals, making it invaluable for applications like speech analysis, fault detection in machinery, and also studying brain activity. At its core, VMD works in an iterative manner by optimizing a mathematical objective function (Dragomiretskiy and Zosso 2013). IMF can be written as a amplitude-modulated-frequency-modulated (AM-FM) signals as

1 $$\begin{aligned} u_k(t) = A_k(t) \cos (\phi _k(t)), \end{aligned}$$

where modal component $u_k(t)$ is a harmonic signal whose amplitude is $A_k(t)$ (non-negative) and instantaneous frequency is $\omega _k(t):=\phi '_k(t)$. Phase $\phi _k(t)$ is an incremental function. Each modal component $u_k(t)$ has a center frequency and a limited bandwidth, and the sum of the modal components is equal to the input signal *f*(*t*).

To obtain the smallest sum of the estimated bandwidths of each component, the constraint variation problem can be described as:

2 $$\begin{aligned} &  \textrm{min}_{\{u_k\},\{\omega _k\}} \bigg \{ \sum _k{\Big \Vert \partial _t \Big [\Big (\delta (t) + j/(\pi t)\Big ) * u_k(t)\Big ] e^{-j\omega _kt} \ \Big \Vert }_2^2\bigg \}, \nonumber \\ &  \text {s.t.}~~ \sum _{k} u_k =f, \end{aligned}$$

where $\{u_k\}:=\{u_1, \ldots , u_K\}$ is the set of variational modal components after decomposition and $\{\omega _n\}:=\{\omega _1, \ldots , \omega _K\}$ is the center frequency set of modal components, $\delta (t)$ is the Dirac function and $*$ denotes convolution. With the objective to find out the best solution for Eq. (2), a quadratic penalty term and Lagrangian multipliers are employed to convert the constrained variational problem into an unconstrained variational problem.

3 $$\begin{aligned} \mathcal {L}(\{u_k\}, \{\omega _k\}, \lambda )&= \alpha \sum _k {\Big \Vert \partial _t \Big [\Big (\delta (t) + j/(\pi t)\Big ) * u_k(t)\Big ] e^{-j\omega _kt} \ \Big \Vert }_2^2 \nonumber \\&\quad + {\Big \Vert f(t)- \sum _k u_k(t)\Big \Vert }_2^2 + {\Big \langle \lambda (t), \ \ f(t) - \sum _k u_k(t) \Big \rangle }, \end{aligned}$$

where $\lambda (t)$ is Lagrange multiplier operator and $\alpha$ is a secondary penalty factor. The original minimization (3) is resolved by identifying the augmented Lagrangian’s saddle point through a series of iterative sub-optimizations known as the alternate direction method of multipliers (Hestenes 1969; Rockafellar 1973). Consequently, component $u_k(t)$ is given by (Dragomiretskiy and Zosso (2013), Algorithm 2):

4 $$\begin{aligned} \hat{u}_k^{n+1}(\omega )=\frac{\hat{f}(\omega ) - \sum _{i< k} \hat{u}^{n+1}_i(\omega )- \sum _{i> k} \hat{u_i}^{n}(\omega ) + {\hat{\lambda }^n(\omega )/2}}{1+2\alpha (\omega -\omega ^n_k)^2}, \end{aligned}$$

identified as a Wiener filtering of the current residual, and the frequency center $\omega _k$ is:

5 $$\begin{aligned} \omega _k^{n+1} = \frac{\int _0^{\infty }\omega \big |\hat{u}^{n+1}_k(\omega )\big |^2d\omega }{\int _0^{\infty } \big |\hat{u}_k^{n+1}(\omega )\big |^2d\omega }. \end{aligned}$$

For all the analysis purpose, a five-IMF decomposition tactic of EEG signals was considered. With the aim to extract the features, a sliding temporal window with a 50% overlap was considered to segment the EEG signal, given that human emotions typically persist for duration ranging from 0.5 to 4 s (Duan etal. 2013). Within each window, the signal was broken down into its different 5 modes using VMD. The Fourier transform was then applied to each of the signal’s decomposed forms, and the power was calculated. After doing so far, power from the predefined frequency bands, specifically Delta (0.5–4 Hz), Theta (4–8 Hz), Alpha (8–12 Hz), Beta (12–30 Hz), and Gamma (30 Hz and above) were extracted which were later used as features for training. The statistical power of the study was calculated by equation (6), which evaluates the power of the decomposed IMFs within specific frequency bands.

6 $$\begin{aligned} P_k = \frac{1}{N} \sum _{n=1}^{N} |X_k[n]|^2, \end{aligned}$$

where $P_k$ represents the power of the *k*th decomposed mode within a specific frequency band, $X_k[n]$ is the Fourier transform of the *k*th mode within the sliding window, and *N* is the total number of samples within the sliding window.

### Machine learning classifiers

Four machine learning classifiers are briefly described below, utilized in various studies by many researchers (Sheth etal. 2022; Abdali et al. 2023; An et al. 2023). These classifiers are utilized and compared herein.

#### K-nearest neighbors (KNN)

KNN is a supervised non-parametric learning classifier (Cover and Hart 1967). KNN is based on proximity or space closeness to make classification about the grouping of data point, where the proximity is commonly based on Euclidean distance (Abu Alfeilat et al. 2019). KNN uses the majority voting criterion, i.e., an object is assigned to a class that is the most common among its *k* nearest neighbors, where the selection of *k* has a significant impact on the learning performance. An optimal value for *k* would reduce the noise effect on the classification while it maximizes the boundary distinction between classes. KNN is easy to implement and understand and has no assumption on the underlying data; however, it has a high computational cost and sensitive to the choice of *k* and distance metric.

#### Support vector machine (SVM)

SVM is a supervised learning classifier used for both linear and non-linear problems (Cortes and Vapnik 1995). In SVM, a hyperplane is computed where the data are virtually transformed to separate classes, and a test point is labeled based on its location to the hyperplane. The points closest to the hyperplane are the support vectors, and the distance between the hyperplane and support vectors define a margin (Awad and Khanna 2015). In SVM, a large margin is considered good. SVM is effective for high-dimensional data; however, it is sensitive to noise and outliers.

#### Decision tree (DT)

DT is a supervised learning classifier, commonly used for non-linear classification problems (Quinlan 1986). DT classifies the data through building a decision tree by very simple binary rule sets. DT has decision nodes which contain conditions to split the data and leaf nodes, helping to decide the class of new data point. The conditions in DT are recursively examined, and the ones maximizing the information gain will be selected. DT is intelligible and very simple to understand and interpret; however, it is prone to overfitting (Sheth etal. 2022).

#### Random forest (RF)

RF is an ensemble supervised learning classifier for solving non-linear classification problems (Breiman 2001). RF comprises many random forests, where each forest contains numerous trees. RF has two random processes, namely, boot strapping and random feature selection. The bootstrapping ensures distinct data for every tree, making the classifier less sensitive to the original training data. The random feature selection helps to reduce the correlation between the trees. Selecting all the features for every tree makes very similar decision nodes, increasing the the similarity between trees for decision making. An ideal size of random features for each tree is close to the square root of the total number of features. As described, RF has a more complex structure compared to DT. However, this complexity is aimed to prevent the overfitting issue, making RF a more robust classifier (Hastie etal. 2009).

## Results

**Fig. 4:**
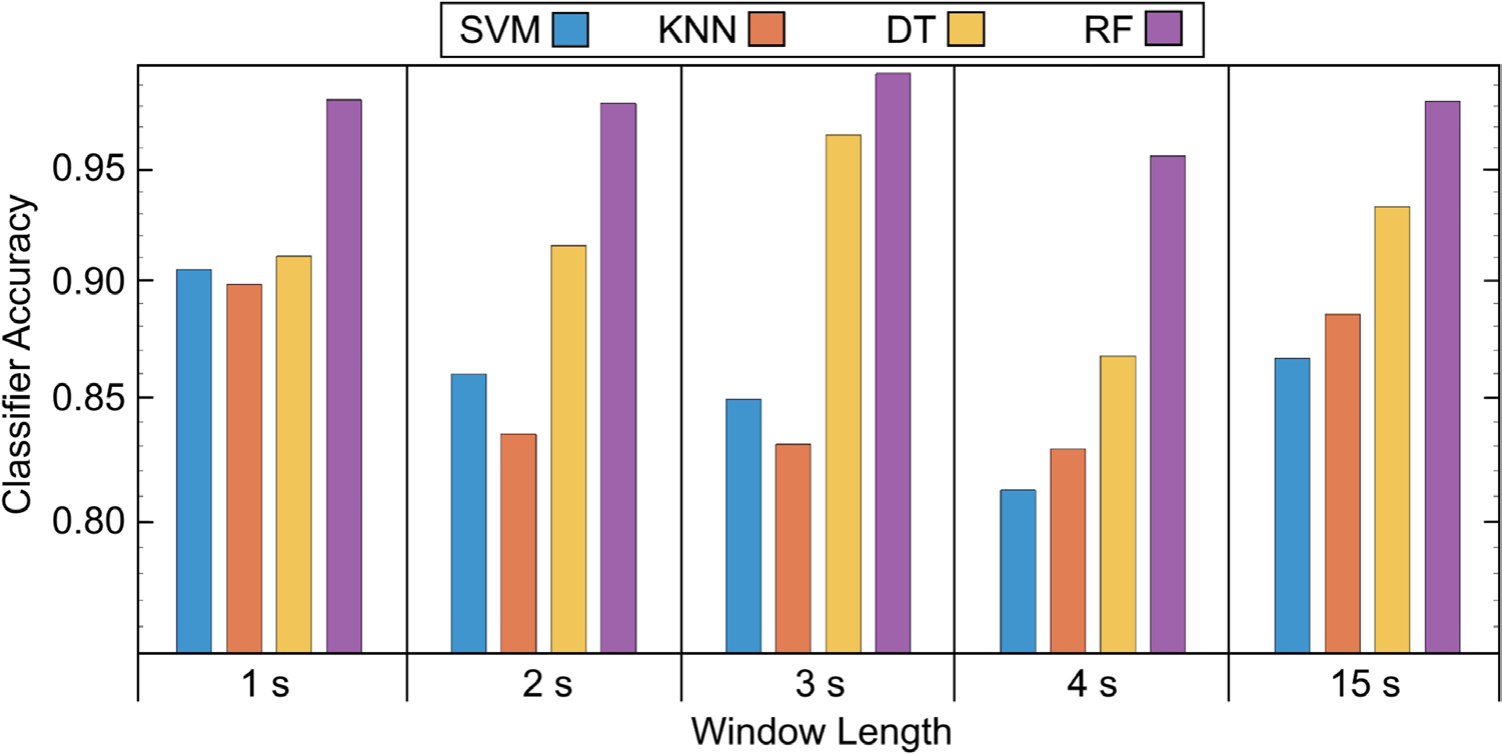
Validation accuracies of different classifiers with respect to different window lengths

**Fig. 5:**
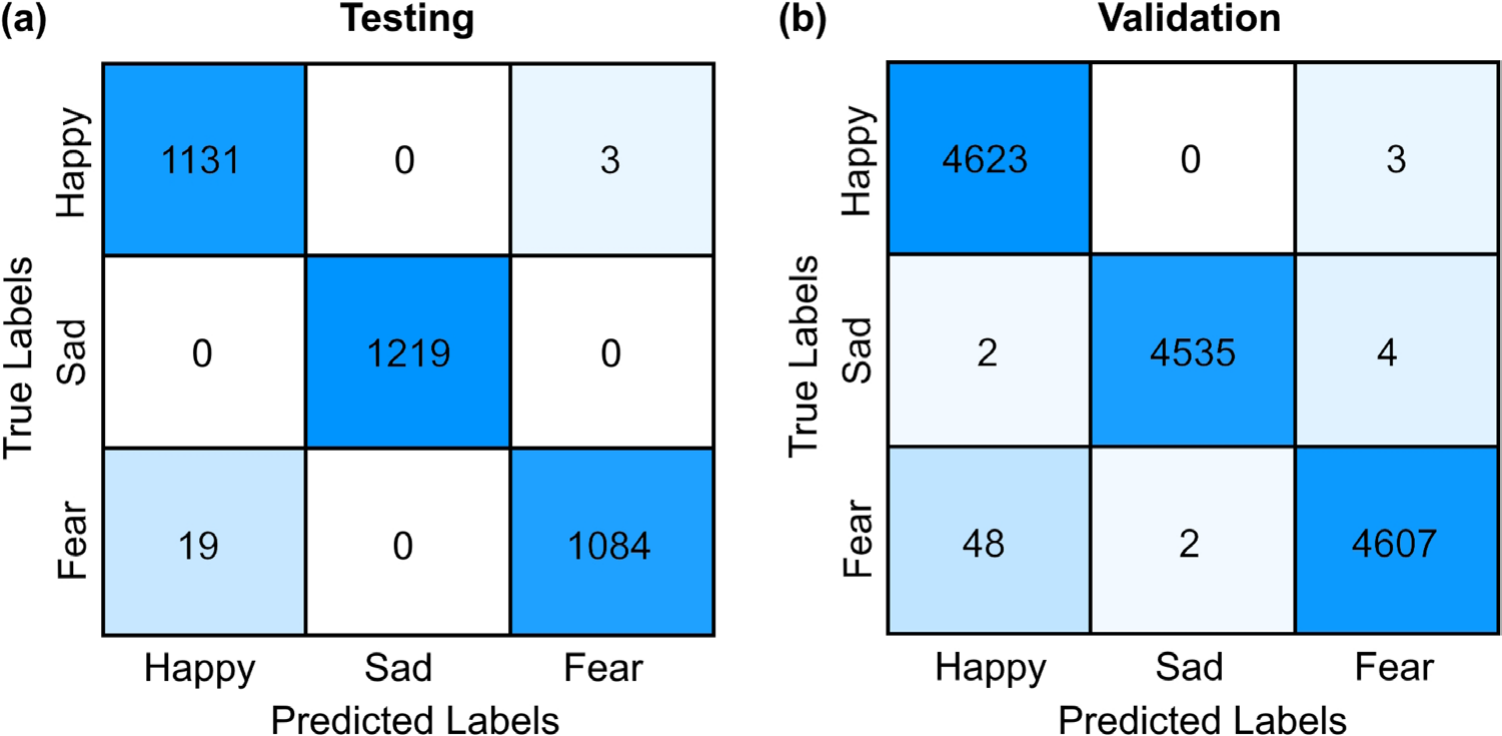
Confusion matrices for RF classifier on the 3-s window

To find the ideal window size and assess the efficacy of various frequency bands for each IMF, a series of classical machine learning classifiers were systematically employed in a repetitive manner. Window lengths 1, 2, 3, 4 s and the whole signal, i.e., 15 s were considered for the experiment. The KNN, SVM, DT, RF were employed for classification purposes. The band-powers of all the frequency bands for all the IMFs were combined together and classified. A train-test split of 80% and 20% was used for the classification tasks. The validation accuracy of each model was evaluated on the training set using a k-fold cross-validation procedure with 6 folds, ensuring robust model assessment and generalization. The trained models were then tested on their respective 20% unseen data. The validation results are shown in Fig. 4 which demonstrates that the tree-based classifiers consistently outperformed other classifiers across the experiment. Furthermore, the RF classifier, employing concatenated frequency features, demonstrates exponential performance improvement, particularly with a window length of 3 s and 50% overlap, achieving a maximum validation accuracy of 99.57% and testing accuracy of 99.36% with a precision of 99.37%, recall of 99.36% and F1 score of 99.36%. The confusion matrices of testing and validation sets are illustrated in Fig. 5.

The best machine learning classifier, i.e., the RF classifier, was applied to the features extracted using the optimal window size for investigating the association of different band powers and IMFs in the classification of emotions. In this experiment, firstly, the band-powers of the five frequency bands from each of five IMFs were examined separately for classification purposes. Secondly, the band-powers of same frequency band of every IMF were concatenated and classified accordingly. Thirdly, band-powers for the same IMFs were concatenated and classified. The validation accuracy for each IMF and each frequency band is listed in Table 1.

**Table 1:** Validation accuracies of IMFs for frequency bands

Signal	Delta	Theta	Alpha	Beta	Gamma	Combined features
IMF 1	0.3733	0.4286	0.3630	0.3784	0.3675	0.5577
IMF 2	0.4520	0.3834	0.3298	0.3365	0.3448	0.8636
IMF 3	0.4209	0.3948	0.3663	0.3454	0.3928	0.7434
IMF 4	0.3996	0.3320	0.5546	0.3528	0.3574	0.8136
IMF 5	0.552	0.3616	0.3776	0.3445	0.3910	0.9573
All IMFs	0.9747	0.8254	0.7828	0.5466	0.6207	**0**.**9957**

The value in bold (0.9957 or 99.57%) is the best validation accuracy obtained among all the experiments

**Fig. 6:**
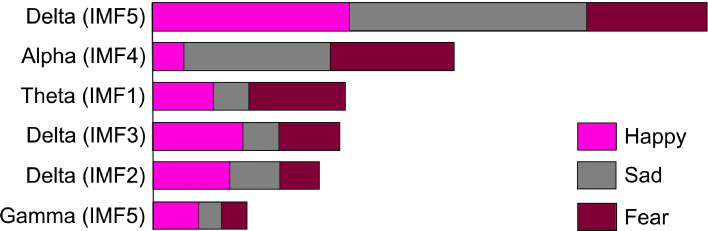
Average SHAP values

To better understand the trained best classifier model and increase its transparency, SHAP (SHapley Additive exPlanations) tool was implemented. The SHAP summary plot, illustrated in Fig. 6, delineated the top six influential features driving emotion prediction out of 25 features. These are identified as Delta band power of IMF 5, Alpha band power of IMF 4, Theta band power of IMF 1, Delta band power of IMF 3, Delta band power of IMF 2, and Gamma band power of IMF 5. No IMF of Beta band comes in the top 6 influential features.

The IMFs provide insights into relevant neurophysiological mechanisms of emotion processing by reflecting distinct frequency-specific contributions linked to emotional states. Table 1 and the SHAP plots demonstrate that specific frequency bands within IMFs are strongly associated with different emotional conditions. Also, IMF 5 has the highest association with emotion processing among all IMFs followed by IMF 4.

In the present study, the SHAP analysis underscored the importance of certain IMFs in predicting specific emotions. For instance, the Delta band power in IMF 5 showed a substantial contribution to the “sad” emotional state, suggesting that it reflects low-frequency neural oscillations tied to emotional processing mechanisms. Similarly, the Alpha band power in IMF 4, contributed to emotion prediction, may point to regions engaged in reduced stress and attentiveness.

## Discussion

The research conducted in this study entails a detailed analysis of EEG signals in the frequency domain employing VMD for the purpose of classifying evoked emotions. The present study demonstrated the effectiveness of VMD in decomposing EEG signals into IMFs, revealing meaningful frequency domain features linked to diverse emotional states. Specific frequency ranges were identified within these IMFs that exhibit distinct patterns across emotional categories, facilitating the development of reliable machine learning models for multi-class emotion classification.

The analysis revealed compelling evidence highlighting the significance of certain features in identification of emotions from EEG signals. Specifically, Table 1 showed that when the ML model was trained solely on extracted features of IMF 5, it yielded higher validation accuracy compared to all other IMFs, indicating the importance of IMF 5 in this context. Similarly, superior accuracy was achieved when focusing exclusively on the Delta band power of all combined IMFs, underscoring the pivotal role of Delta band power in emotion recognition with EEG signals’ features in the frequency domain obtained using VMD. Furthermore, the highest accuracy was achieved with features of all IMFs combined.

The SHAP results, depicted in Fig. 6, has uncovered that the Delta band power of IMF 5 exhibited a significant contribution towards predicting the “sad” emotional class. Conversely, its influence on the “happy” class was smaller and even less on the “fear” class. Similar patterns were observed for the Alpha band power of IMF 4. In contrast, the Theta band power of IMF 1 was associated with a higher likelihood of the model classifying into a “fear” emotional state. Interestingly, Delta band power of IMF3 and IMF2 showed a significant association with “happy” and a smaller association with “sad” and “fear”. Interestingly, the Delta band power of IMF 3 and IMF 2 showed a high contribution in predicting into a “happy” emotional state by the model. Also, no IMF of Beta band came in the top 6 influential features.

The present study demonstrated superior classification accuracy in three-class emotion classification compared to previous studies (Li and Lu 2009; Wang et al. 2014; Mehmood and Lee 2015; Guo and Wang 2024). Wang et al. (2014) achieved an average accuracy of 87.53% among 6 subjects distinguishing positive and negative emotions using EEG power spectra and SVM. Li and Lu (2009) achieved 93.5% accuracy (testing) classifying “happy” and “sad” emotions with logarithm variance of EEG trials and SVM. Mehmood and Lee (2015) addressed “happy”, “sad”, “scared”, and “calm” emotions with 61% accuracy using KNN and Hjorth parameters. Guo and Wang (2024) classified multiple emotions with 94.22% accuracy using CGRU-MDGN.

The comparison above highlights the superior performance of the proposed method in accurately classifying the three evoked emotions, demonstrating its effectiveness in the field of EEG-based emotion classification. The present study, however, is limited by a small sample size, which may affect the statistical power and generalizability of the findings. Additionally, the study focuses on the classification of only three emotion classes and involves offline analysis of the frequency domain features in the temporal domain. The analysis does not cover the spatial domain features of EEG data. Future work will address these limitations by including a larger sample size, expanding the research to cover additional emotion classes, and exploring methods for real-time analysis.

## Conclusions

The research work explored the utilization of VMD alongside frequency domain properties of each IMF to analyze EEG signals for emotion recognition. The findings elucidate optimal window lengths and underscore the substantial contributions of individual IMFs and frequency ranges in predicting emotions. Furthermore, through a comparative analysis of various machine learning models, the superior efficacy of the random forest classifier in emotion recognition tasks is highlighted. This classifier’s robust performance underscores its ability to accurately classify emotional states from EEG signals. Moreover, the study used the Shapley additive explanations (SHAP) values to augment interpretability by establishing the relationship between model predictions and input features. SHAP analysis elucidates the extent to which each feature influences predictions for individual instances, facilitating a more detailed understanding of the model’s decision-making process. This aspect is particularly crucial for applications involving high-risk scenarios, such as emotion recognition. Overall, the work underscores the potential of integrating signal decomposition techniques and machine learning models for classifying emotions from brain signals. The insights gleaned hold significant implications for future research endeavors in affective computing, mental healthcare assessment, and human–computer interaction, promising advancements in understanding and addressing emotions in various domains.

## Data Availability

The data used in this research can be made available upon a reasonable request.

## Abbreviations

AM-FM: Amplitude-modulated-frequency-modulated
CMS: Common mode sense
CNN: Convolutional neural network
DNN: Deep neural network
DRL: Driven right leg
DT: Decision tree
ECG: Electrocardiogram
EEG: Electroencephalogram
EMD: Empirical mode decomposition
FIR: Finite impulse response
ICA: Independent component analysis
IMF: Intrinsic mode function
KNN: K-nearest neighbors
ML: Machine learning
RF: Random forest
SHAP: SHapley Additive exPlanations
SVM: Support vector machine
VMD: Variational mode decomposition
WPD: Wavelet packet decomposition
